# Spatial arrangement, polarity, and posttranslational modifications of the microtubule system in the *Drosophila* eye

**DOI:** 10.1007/s00441-024-03914-6

**Published:** 2024-08-17

**Authors:** Piotr Kos, Otto Baumann

**Affiliations:** Unit of Animal Physiology, Institute of Biochemistry and Biology, University of Potsdam, 14476 Potsdam, Germany

**Keywords:** MTOC, Microtubule polarity, Posttranslational modification, Photoreceptor, Drosophila melanogaster

## Abstract

We have analyzed the organization of the microtubule system in photoreceptor cells and pigment cells within the adult *Drosophila* compound eye. Immunofluorescence localization of tubulin and of Short stop, a spectraplakin that has been reported to be involved in the anchorage of microtubule minus ends at the membrane, suggests the presence of non-centrosomal microtubule-organizing centers at the distal tip of the visual cells. Ultrastructural analyses confirm that microtubules emanate from membrane-associated plaques at the site of contact with cone cells and that all microtubules are aligned in distal–proximal direction within the photoreceptor cells. Determination of microtubule polarities demonstrated that about 95% of the microtubules in photoreceptor cells are oriented with their plus end in the direction of the synapse. Pigment cells in the eye contain only microtubules aligned in distal–proximal direction, with their plus end pointing towards the retinal floor. There, two populations of microtubules can be distinguished, single microtubules and bundled microtubules, the latter associated with actin filaments. Whereas microtubules in both photoreceptor cells and pigment cells are acetylated and mono/bi-glutamylated on α-tubulin, bundled microtubules in pigment cells are apparently also mono/bi-glutamylated on β-tubulin, providing the possibility of binding different microtubule-associated proteins.

**Supplementary Information:**

The online version contains supplementary material available at 10.1007/s00441-024-03914-6.

## Introduction

Photoreceptor cells (PRCs) in the *Drosophila* compound eye are highly polarized neuroepithelial cells. They are organized into several compartments and membrane domains that differ by function and protein equipment (Satoh et al. 2013). The light-receptive portion of the PRC, the rhabdomere, is a column of densely packed microvilli that runs alongside the soma and houses the molecular components of the phototransduction machinery (Katz and Minke 2009). The remaining soma executes household functions, such as protein synthesis and maintenance of ion gradients, whereas the synapse contains proteins for target recognition and signal transmission (Tong et al. 2011; Xu et al. 2015; Schopf and Huber 2017).

*Drosophila* PRCs are used as a model system for analyzing the mechanisms involved in the targeted delivery of membrane proteins to distinct membrane domains (Tong et al. 2011; Muschalik and Knust 2011; Schopf and Huber 2017). This function involves sorting of molecules into distinct populations of transport vesicles, a process that occurs at the trans-Golgi network (TGN; Satoh et al. 2013; Iwanami et al. 2016; Nakamura et al. 2020). Subsequently, the various vesicle populations are delivered to the proper subcellular compartment via directional transit routes in the cytoplasm (Li et al. 2007; Iwanami et al. 2016; Nakamura et al. 2020). The latter task is accomplished by polarized cytoskeletal elements in conjunction with motor proteins that transport their load in a unidirectional mode along cytoskeletal rails.

The microtubule (MT) cytoskeleton plays a chief role in the establishment of cell polarity (Baas and Lin 2011; Akhmanova and Kapitein 2022). MTs are composed of α/β-tubulin heterodimers that form tube-like polymers with a fast-growing plus end and a slow-growing minus end. The latter end is usually situated at a microtubule-organizing center (MTOC) that serves for MT nucleation. MTOCs are either centrosomes, two centrioles surrounded by an amorphous pericentriolar material, or, in differentiated cells, non-centrosomal structures (ncMTOCs; Lüders and Stearns 2007; Toya and Takeichi 2016; Sanchez and Feldman 2016). Polarized positioning of the MTOC within the cell and polar construction of MTs, with the minus-end centered at the MTOC and the plus end pointing away from the MTOC, provide a structural framework for directional transport and polarized secretion. For adult *Drosophila* PRCs, however, neither identity nor position of the MTOC nor MT polarity has been clarified unequivocally (Tillery et al. 2018; Laffafian and Tepass 2019). But this information is fundamental for the interpretation of phenotypes of mutations in microtubule-associated proteins (MAPs), including MT motor proteins.

Since developing PRCs lose their centrioles during pupal life (Riparbelli et al. 2018), it may be presumed that in adult PRCs, MT organization occurs via ncMTOCs. The nucleus of adult PRCs, like in developing PRCs, is located at a short distance below the distal end of the cell, and MTs occur both distally and proximally of the nucleus (Górska-Andrzejak et al. 2003). Since the MT system proximal of the nucleus extends into the axon, it may be presumed that these MTs have uniform polarity with their plus end directed to the synapse, as in other *Drosophila* neurons (Stone et al. 2008). This assumption is in line with the finding that mutants in Milton, a protein associated with the plus-end-directed MT motor protein kinesin, have defective axonal transport of mitochondria towards the synapse (Stowers et al. 2002). Moreover, such a MT orientation in adult PRCs corresponds to MT orientation in epithelial cells of the larval eye disc (Fernandes et al. 2014). Apart from that, mixed MT polarity has been reported for adult PRCs of another arthropod, the mantis shrimp (King and Cronin 1993). With respect to MTs above the nucleus, the situation is even less clear, and there are several possible scenarios for their orientation. (1) MTs may have their plus end oriented away from the nucleus, as suggested for pupal photoreceptors (Mosley-Bishop et al. 1999; Patterson et al. 2004). (2) MT minus ends may point away from the nucleus, similar to the situation in dendrites of *Drosophila* sensory neurons (Stone et al. 2008). (3) A mixed MT polarity may occur above the nucleus, similar to vertebrate dendrites or stomatopod PRCs (Baas and Lin 2011; King and Cronin 1993).

Here, we show that adult *Drosophila* PRCs have membrane-associated ncMTOCs at their distal end, viz. the junction with cone cells. Over the entire length of the visual cell, MTs have the same orientation, with the vast majority having their plus end pointing towards the synapse. We also analyze the organization of the MT cytoskeleton in 2°/3° pigment cells (PCs), showing that these contain two different sets of MTs, single MTs and MT bundles, both extending in distal–proximal direction and of uniform polarity, with the plus ends pointing towards the retinal floor. Finally, we demonstrate that MT systems of the various cell types differ in posttranslational modifications, suggesting differences in stability and interaction with microtubule-associated proteins (MAPs). These results provide a basis for future studies on MT-dependent protein and vesicle trafficking in adult *Drosophila* PRCs.

## Materials and methods

### Animals

Flies were reared on standard corn meal medium at 22 °C under a 12-h light/dark rhythm. Oregon-R flies marked with *w*^*1118*^ and the green fluorescent protein (GFP)-tagged Atpα line *Atpα*^*G00109*^ (Bloomington Drosophila Stock Center, Indiana University, Bloomington, IN, USA) were used for immunofluorescence microscopy; *w*^+^ flies were used for transmission electron microscopy. *UAS-nod::lacZ* (courtesy of Y. N. Jan; Clark et al. 1997) and *elav-Gal4* (Bloomington Drosophila Stock Center) were used for probing MT polarity.

### Immunofluorescence microscopy

Eyes were dissected and fixed as described previously (Baumann 2004). After extensive washing with 0.1 M phosphate buffer (pH 7.0), specimens were sequentially infiltrated with phosphate-buffered 10% sucrose (30 min at room temperature) and 25% sucrose (overnight at 4 °C), embedded in a thin layer of phosphate-buffered 2% low-melting agarose supplemented with 25% sucrose, and frozen in melting isopentane. Ten-micrometer-thick sections were cut on a cryostat and labeled with primary antibodies (Table 1) as described in detail previously (Baumann 2004).

**Table 1:** List of primary antibodies used in this study. *M* mouse monoclonal, *R* rabbit polyclonal, *IF* immunofluorescence, *WB* Western blotting

Antigen	Raised in…; clone	Supplier	Dilution for IF	Dilution for WB	Reference
α-Tubulin	M; DM1A	Sigma-Aldrich Chemie GmbH, Munich, Germany	5 µg/ml	0.5 µg/ml	Blose et al. (1984)
β-Tubulin	M; E7	Developmental Studies Hybridoma Bank (DSHB), University of Iowa, Iowa City, Iowa	1 µg/ml	0.1 µg/ml	Klymkowsky (1989)
Acetylated tubulin	M; 6-11B-1	Sigma-Aldrich	0.5 µg/ml	0.05 µg/ml	Piperno and Fuller (1985)
Tyrosinated tubulin	M; TUB-1A2	Sigma-Aldrich	1.5 µg/ml	0.15 µg/ml	Kreis 1987
Detyrosinated tubulin	M; 1D5	Synaptic Systems, Göttingen, Germany	10 µg/ml	1.0 µg/ml	Rüdiger et al. (1999)
Polyglutamylated tubulin	M; GT335	Bernard Eddé, Montpellier, France	1:1,000	1:10,000	Wolff et al. (1992)
Polyglutamylated tubulin	M; B3	Sigma-Aldrich	40 µg/ml	4.0 µg/ml	Gagnon et al. (1996)
*Drosophila* Shot	M; mAbRod1	DSHB	5 µg/ml		Kolodziej (2003)
Na,K-ATPase α-subunit	M; α5	DSHB	10 µg/ml		Lebovitz et al. (1989)
β-Galactosidase	M; 40-1a	DSHB	3.6 µg/ml		Ghattas et al. (1991)
GFP	R; -	Thermo Fisher Scientific GmbH, Dreieich, Germany	1:400		

Cy3-conjugated goat anti-mouse IgG, Cy3-conjugated goat anti-rabbit IgG, Cy5-conjugated goat anti-mouse IgG, Cy5-conjugated goat anti-rabbit IgG (Jackson ImmunoResearch Europe Ltd., Newmarket, Suffolk, UK), AlexaFluor 568-labeled goat anti-mouse-IgG, AlexaFluor 546-labeled goat anti-mouse-IgG2b, and AlexaFluor 633-labeled goat anti-mouse-IgG1 (Thermo Fisher Scientific, Waltham, MA, USA) were used as secondary antibodies. AlexaFluor 488-phalloidin and 4′,6-diamidino-2-phenylindole (DAPI) were added as labels for actin filaments and DNA, respectively. Sections were analyzed with LSM510, LSM510-Meta, or LSM710 confocal microscopes (Carl Zeiss Microscopy GmbH, Jena, Germany).

### Electron microscopy

Fly heads were bisected and immersed in fixative (2% glutaraldehyde, 3% paraformaldehyde in 0.1 M Na-cacodylate buffer, pH 7.4). After 1 h at room temperature, specimens were transferred to fixative supplemented with 1% tannic acid, incubated for an additional hour, washed 3 × 10 min in 0.1 M cacodylate buffer, and post-fixed for 30 min in 2% cacodylate-buffered OsO_4_. After extensive washing in H_2_O, samples were dehydrated in a graded ethanol series and acetone and embedded in Spurr resin by standard procedures. Sections were cut at 70 nm thickness, contrasted with uranyl acetate and lead citrate, and examined in a Philips CM100 electron microscope, operated at 80 kV.

### Decoration of microtubules with exogenous tubulin

Microtubule polarity assay was performed according to the protocol of Troutt and Burnside (1988). Bisected *Drosophila* heads were incubated for 30 min on ice in 1% Triton X-100, 0.5% sodium deoxycholate, 0.2% sodium dodecyl sulfate (SDS), 2.5% dimethylsulfoxide, 1 mM MgCl_2_, 1 mM K_2_ ethylene glycol-bis(β-aminoethyl ether)-N,N,N′,N′-tetraacetic acid, 1 mM guanosine-5′-triphosphate, and 0.5 M piperazine-N,N′-bis(2-ethanesulfonic acid) (pH 6.9), containing 1.8 mg/ml bovine brain tubulin (Cytoskeleton, Denver, CO, USA). Temperature was raised to 22 °C for 15 min and then to 37 °C for 15 min to promote polymerization of tubulin. Subsequently, specimens were fixed and analyzed by transmission electron microscopy as described above.

## Results

### Spatial arrangement of microtubules in the retinal cells

**Fig. 1:**
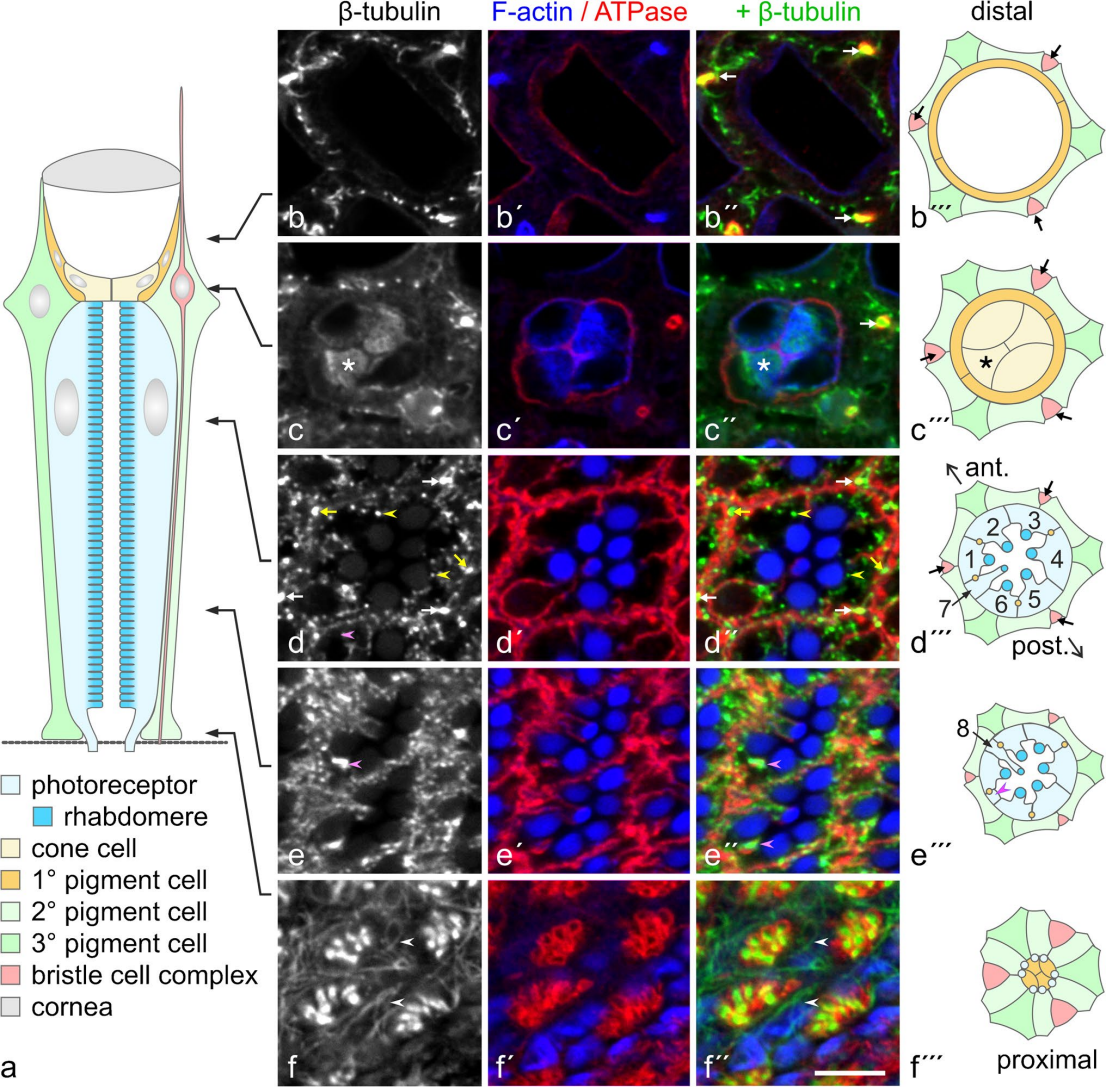
Immunofluorescence localization of microtubules (MTs) in adult *Drosophila* eyes. Cryo-sections at various levels (level indicated in **a**) through eyes of *ATPα*::*GFP* flies were labeled with anti-β-tubulin (green in **b″**, **c″**, **d″**, **e″**, **f″**), AlexaFluor 488 phalloidin (blue in **b″**, **c″**, **d″**, **e″**, **f″**) and anti-GFP (red in (**b″**, **c″**, **d″**, **e″**, **f″**). Schematic drawings on the right (**b′″**, **c′″**, **d′″**, **e′″**, **f′″**) indicate the cell pattern in a cross-sectioned ommatidium according to the level of the individual cryo-section, and with the anterior–posterior orientation (ant./post.) similar to the cryo-section. Note that for simplicity, the bristle cell complex is not further subdivided into its individual cells (bristle neuron, supplementary cells) in the schemes. Na,K-ATPase/GFP labeling (ATPase) outlines bristle neurons (white arrows in **b″**, **c″**), cone cells (**c″**), and photoreceptor cells (**d″**, **e″**, **f″**). In cone cells, staining for tubulin (asterisk in **c**, **c″**, **c′″**) colocalizes with F-actin. Bristle neurons (white arrows), as identified by their characteristic position in an ommatidium (black arrows in **b′″**, **c′″**, **d′″**), are intensely labeled over their entire length for tubulin, indicating a MT array. Tubulin-positive structures between photoreceptors (yellow arrows in **d**, **d″**) and of similar intensity to bristle neurons are indicative of MT bundles in 2° and 3° pigment cells. Dot-like tubulin-positive structures in photoreceptors (yellow arrowheads in **d**, **d″**) represent longitudinally aligned MTs. Magenta arrowheads in (**e**, **e″**, axons of R7 photoreceptors. White arrowheads in **f**, **f″**), MTs en-face-view in pedicels of 2° and 3° pigment cells between bundles of photoreceptor axons. Bar, 5 µm

The spatial arrangement of MTs in the adult *Drosophila* eye was probed by immunofluorescence microscopy with anti-β-tubulin and by transmission electron microscopy. Figure 1(a) presents in a schematic mode the various cell types of an ommatidium in the eye. To visualize the outline of these cells in anti-tubulin-stained sections, we wanted to co-label for Na,K-ATPase (Baumann et al. 2010). Since co-staining with anti-β-tubulin and anti-Na,K-ATPase α-subunit was impossible as both antibodies were of the same species and isotype, immunofluorescence microscopy was carried out on *ATPα*::*GFP* flies that express GFP-tagged Na,K-ATPase α-subunit. It should be noted however that identical results were obtained on wildtype flies with anti-acetylated α-tubulin and anti-Na,K-ATPase α-subunit (Supplementary Material, Fig. S1).

#### Bristle neurons

Bipolar bristle neurons are composed of an outer and an inner dendritic segment, a soma and an axon. The MT system of the outer dendritic segment, a modified cilium, has been described before (Mogensen et al. 1993) and is typical for an insect mechanoreceptor (Supplementary Material, Fig. S2; Chi and Carlson 1976; Keil 1997). Notably, the connection between the outer and inner segment is the only site in the retina with a centriole. Distally of the basal body, the cilium contains irregularly arranged single MTs and MT pairs that extend into a tubular body of closely packed MTs at the distal tip of the outer dendritic segment. The inner dendritic segment and the axon contain few MTs, widely spaced at 50–200 nm. In immunofluorescence images, bristle neurons were identified by their characteristic position between ommatidia and by intense staining for tubulin over the entire length (Fig. 1b–b′″, c–c′″, d–d′″; Supplementary Material, Fig. S1 b-b′″, c–c′″, d-d′″).

#### Cone cells

The spatial organization of MTs in *Drosophila* cone cells has been analyzed in detail previously by transmission electron microscopy. Cone cells contain numerous, closely packed but irregularly spaced MTs that extend in apicobasal direction, with both MT ends associated with electron-dense plaques at the plasma membrane (Mogensen et al. 1993). In immunofluorescence images, cone cells appear to be stained almost homogenously for tubulin throughout their entire cytoplasm since MTs are too closely packed to be optically resolved by confocal microscopy (Fig. 1c–c″; Supplementary Material, Fig. S1c-c″).

#### 2° and 3° pigment cells

**Fig. 2:**
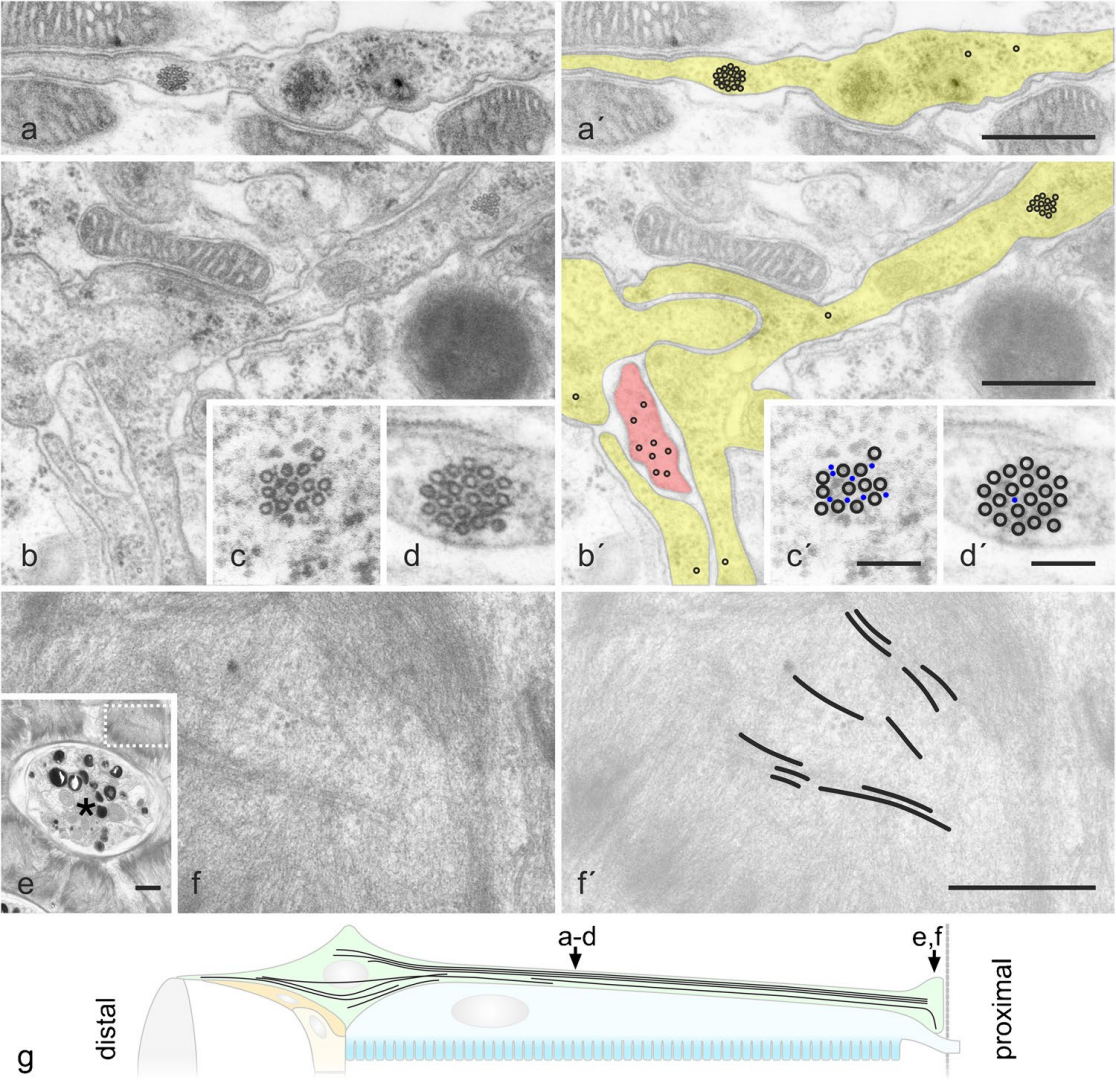
Organization of the microtubule (MT) system in 2° and 3° pigment cells. **a**–**f** Electron micrographs of cross-sections through pigment cells. **g** Location of section plane is indicated in the schematic drawing (color coding corresponding to schemes in Fig. 1). **a′**, **b′**, **c′**, **d′**, **f′** MTs highlighted in pigment cells (yellow in **a′**, **b′**) and in a bristle neuron (red in **b′**). **c**, **d** Pigment cells contain individual MTs and MT bundles. Dot-like structures between bundled MTs (blue in **c′**, **d′**) likely represent actin filaments. Non-colored cells in **a′**, **b′** are photoreceptor cells. **e** The retinal floor composed of bundled photoreceptor axons (asterisk) surrounded by pigment cell pedicels. The dashed rectangle in **e** indicates the pigment cell pedicel shown at higher magnification in **f**, **f′**. MTs are highlighted in **f′**. Bars in **a**, **b**, **e**, **f**, 0.5 µm; in **c**, **d**, 0.1 µm

In immunofluorescence images of retinal cross-sections stained with anti-tubulin, dot-like structures were observed between photoreceptors of adjacent ommatidia, indicating longitudinally arranged MTs in PCs (Fig. 1d″; Supplementary Material, Fig. S1d-d″). Electron microscopy has confirmed a longitudinal orientation of MTs and has visualized two different MT populations in this cell type (Fig. 2a–d, a′–d′). Besides single MTs widely distributed over the entire cross-sectional profile, PCs contained bundles of 4 to 20 MTs (10.04 ± 4.01; mean ± s.d.; *n* = 28). MTs were hexagonally packed in these arrays, keeping a distance of about 10–20 nm, with few individual ~ 5-nm-thick filamentous structures in between, likely actin filaments (Fig. 2c, c′, d, d′). In anti-tubulin fluorescence images, these MT bundles appeared as structures with a labeling intensity similar to the axons of bristle neurons. MT bundles were observed in 2° and 3° PCs between the nucleus and the PC pedicel. Just one bundle was detected per cell, whereby this occupied a relatively central position within the cross-sectional profile. In the pedicels, MTs were oriented parallel to the basal lamina (Figs. 1f–f″ and 2e, f, f′; Supplementary Material, Fig. S1f, f″), suggesting that MTs make a 90° curve as they extend from the thin cell sheet between adjacent retinulae into in the basal portion of pigment cells.

#### Photoreceptor cells

By immunofluorescence microscopy and by electron microscopy, all MTs detected within photoreceptor cells (PRCs) were aligned to the cell’s long axis. MTs were distributed throughout the cytoplasm, being enriched at the basolateral plasma membrane and next to adherens junctions (Fig. 1d–d″; Supplementary Material, Figs. S1d-d″, S3). About 5–10 MTs were observed per cross-sectional profile of an outer (R1–R6) PRC. Although we cannot exclude that some MTs may have escaped detection and/or may have not been preserved by chemical fixation, the MT number and their alignment agree well with findings on high-pressure-frozen, freeze-substituted specimens (Mun et al. 2009).

### Distribution of putative MTOCs in photoreceptor cells

**Fig. 3:**
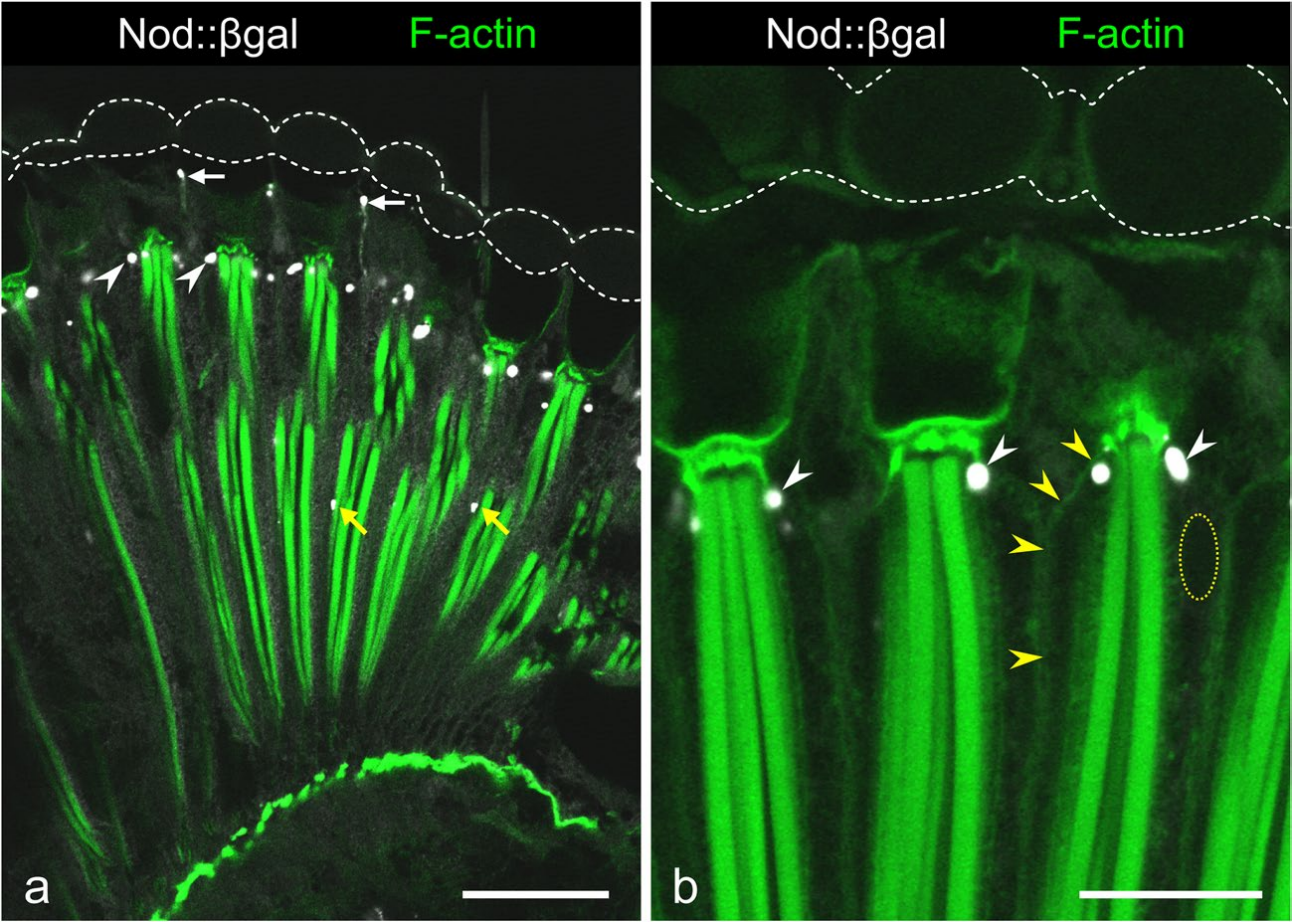
Expression of the microtubule minus-end motor Nod::βgal in sensory neurons in the eye. White arrows, Nod::βgal in bristle neurons; white arrowheads, Nod::βgal at the distal tip of R1-R6 photoreceptor cells; yellow arrows, Nod::βgal likely in R8 cells. For the phalloidin image in **b**, gamma correction was set to 0.65 to visualize structures of faint staining, viz. the peripheral domain of the plasma membrane (yellow arrowheads). White dashed lines delineate the cornea. The yellow dashed circle indicates the position of a photoreceptor nucleus, as deduced from the corresponding differential interference contrast image (not shown). Bar in **a**, 25 µm; in **b**, 5 µm

Since centrioles are absent in the adult eye, except for the basal body in bristle sensory neurons, the question arises on the identity and position of MTOCs in adult PRCs. In order to localize MTOCs, we expressed Nod::βgal by using a *UAS-nod::lacZ* transgene, as performed previously on larval eye discs (Mosley-Bishop et al. 1999; Patterson et al. 2004). Nod::βgal moves towards the MT minus end (Clark et al. 1997). Using an *elav-Gal4* driver, Nod::βgal was expressed in PRCs and bristle neurons of adult eyes. In the latter cells, Nod::βgal was concentrated at a position corresponding to the basal body (Fig. 3a), in accordance with a location of the MT minus ends at the basal body serving as MTOC. In PRCs R1-R7, Nod::βgal was located at the very distal end, just below cone cells (Fig. 3a, b). Finally, Nod::βgal was detected at a position in the retina that corresponds to the distal end of R8 (Fig. 3a).

**Fig. 4:**
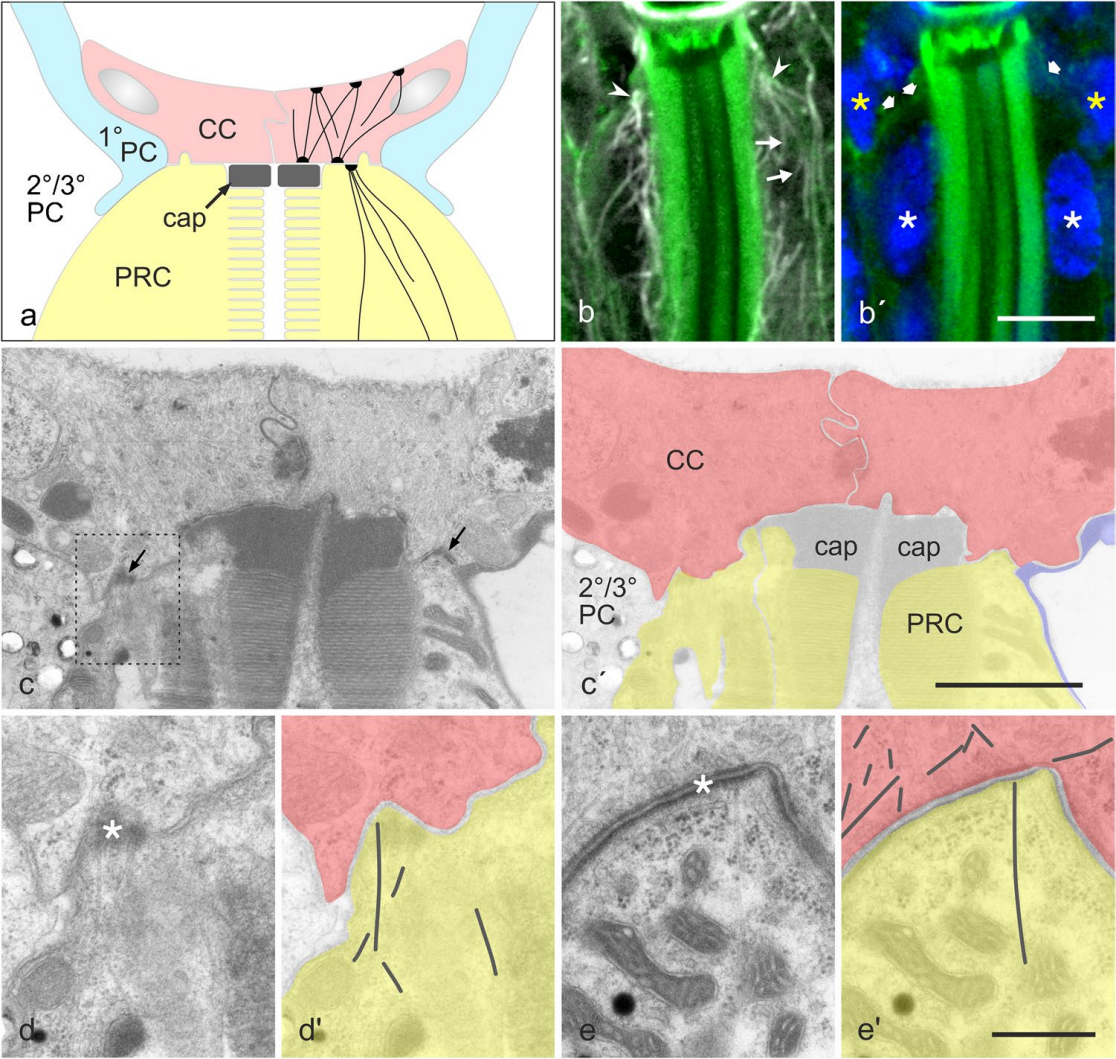
Microtubules (MTs) originate at the distal tip of photoreceptor cells. **a** Schematic drawing of the distal ending of photoreceptor cells (PRC; yellow), attached to cone cells (CC; red), and surrounded by 1° (blue), 2°, and 3° pigment cells (PC). A cap of extracellular material (cap) is positioned between each rhabdomere tip and CCs. Organization of the MT system, as deduced from immunofluorescence and electron microscopy images, is indicated by black lines in CC and PRC. **b**, **b′** Longitudinal cryo-section stained with DAPI (blue in **b′**), AlexaFluor 488 phalloidin (green), and anti-Ac-α-tubulin (white in **b**). In case of phalloidin, gamma correction was set to 0.65 to visualize the peripheral domain of the plasma membrane (broad arrows in **b′**)**.** Several MTs emanate from a focus at the distal tip of the photoreceptor (white arrowheads), extend basally and run aside (thin arrows) the nucleus. Nuclei of photoreceptor cells are indicated by white asterisks, nuclei of pigment cells by yellow asterisks. **c**, **c′** Electron micrograph showing CCs (outlined in red in **c′**) and the distal tip of PRCs (yellow in **c′**). Note the adhesion sites (arrows in **c**) between PRCs and CCs. The dashed rectangle in **c** is presented at higher magnification in **d**. **d**, **e** In PRCs, MTs arise at adhesion sites with CCs. **d′**, **e′** MTs highlighted in PRCs (yellow) and CCs (red). Bar in **b**, 5 µm; in **c**, 2 µm; and in **d**, **e**, 0.5 µm

To identify MTOCs in PRCs by an alternative method, longitudinal sections through eyes were stained with anti-Ac-α-tubulin, DAPI, and phalloidin (Fig. 4b, b′). By serial optical sectioning, we were unable to identify a structure that qualifies as a MTOC in the vicinity of the photoreceptor nucleus. However, about 5 to 10 µm above the nucleus, at the distal end of the visual cell just below the cone cells, several MTs appeared to emanate from a single focus, extended in proximal direction, and bypassed the nucleus. When we examined the distal cell portion by transmission electron microscopy, MTs were detected in PRCs that extended to focal adhesion sites with cone cells, being inserted with one end in an electron-dense cortical matrix (Fig. 4c–e, c′–e′). These findings suggest that adult PRCs have ncMTOCs associated with cortical foci at the distal end of the cell (Fig. 4a). Notably, membrane-associated plaques have been also reported to serve as ncMTOCs in cone cells (Mogensen et al. 1993).

**Fig. 5:**
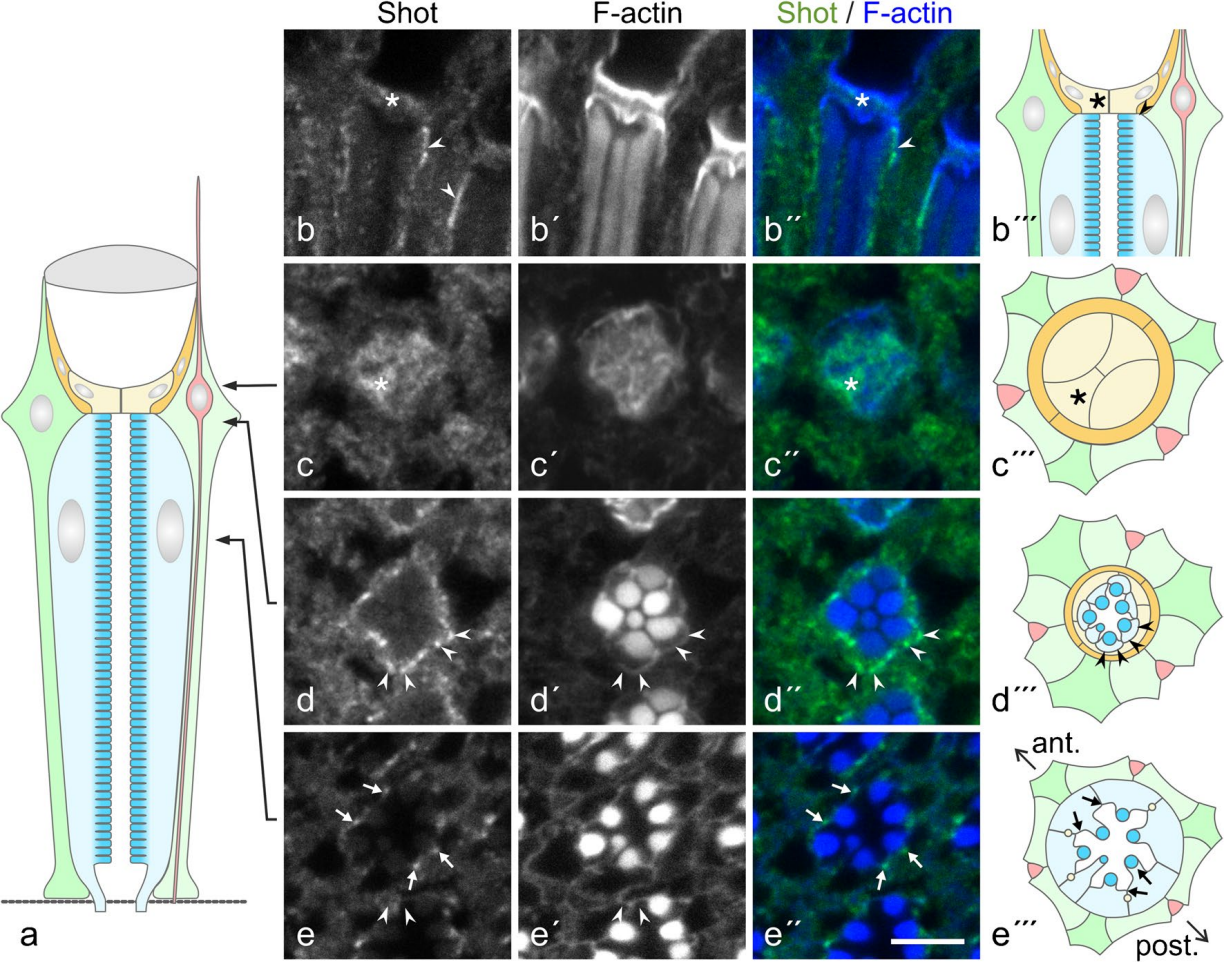
The spectraplakin Short stop (Shot), a cortical anchor for non-centrosomal MTOCs, is enriched at the distal end of photoreceptor cells. **b–e**, **b′–e**′, **b″–e″** Cryo-sections were labeled with anti-Shot and AlexaFluor 488 phalloidin (F-actin). In the case of phalloidin images, gamma correction was set to 0.6 to visualize areas of faint staining, viz. the basolateral domain of PRCs (arrowheads in **d′**, **d″**). **b–b″** The distal portion of ommatidia in longitudinal view. **b–d** Cross-sections through ommatidia at various levels (indicated in **a**). Shot is enriched at the distal end of photoreceptor cells on the basolateral membrane domain (arrowheads in **d**). Moreover, staining for Shot is detected in cone cells (asterisks) and close to the rhabdomere base (arrows). **b′″–e′″** Position of the labels (asterisk, arrowheads, arrows) is also indicated in the schemes. Color coding of schematic drawings (**a**, **b′″–e′″**) corresponds to schemes in Fig. 1. Bar, 5 µm

The study on *Drosophila* oocytes and the fat body suggest that the proteins Short stop (Shot) and Patronin are involved in the formation of ncMTOCs (Nashchekin et al. 2016; Zheng et al. 2020). Shot is a spectraplakin, a huge linker protein with an N-terminal actin-binding domain and two C-terminal MT-binding domains, and recruits the MT-minus-end-binding Patronin to the cell cortex (Zhang et al. 2017). Anti-Shot immunofluorescence on adult eyes demonstrated an enrichment of this protein at the distal end of the PRCs, in association with the plasma membrane (Fig. 5b–b′″, d–d′″). Position of Shot thus coincides with the position of ncMTOCs in PRCs. Weak immunoreactivity for Shot was further detected in PRCs at the rhabdomere base and close to adherens junctions (Fig. 5e–e′″), and in cone cells (Fig. 5b–b′″, c–c′″).

### Microtubule polarity

MT polarity in the various cell types of the adult eye was determined by the hook assay (Heidemann and McIntosh 1980; Baas and Lin 2011). Since the orientation of the samples was maintained from embedding through imaging and since observations were made looking from the corneal side towards the basal lamina, MTs with clockwise-oriented hooks had their plus end facing the observer, while MTs with counterclockwise-oriented hooks had their minus end towards the observer. Bristle neurons served as an internal control, since it is expected that MTs on both sides of the basal body have their minus ends oriented towards this structure.

**Fig. 6:**
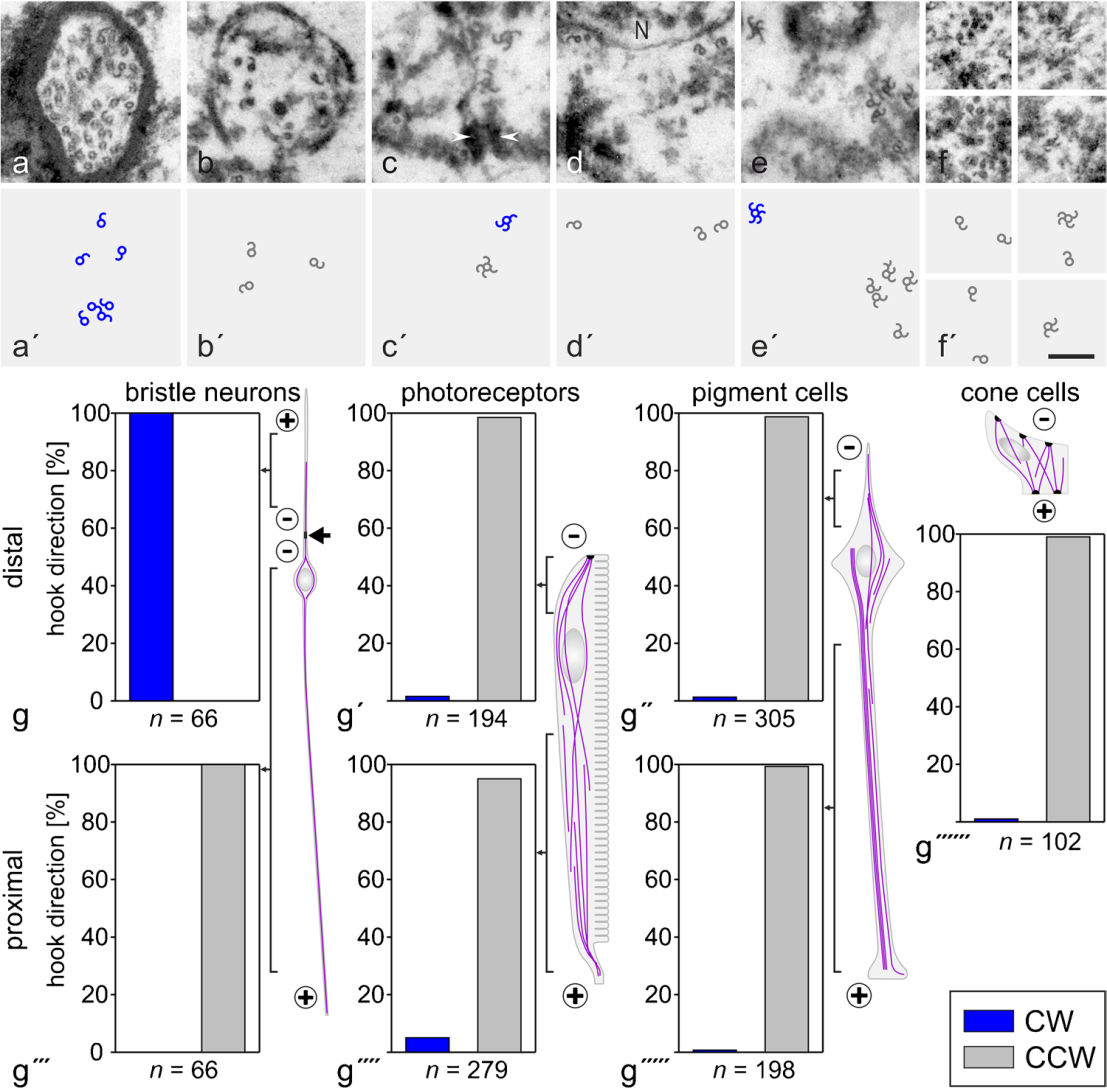
Microtubule (MT) polarity in the *Drosophila* eye as determined by the hook assay. **a** Dendritic outer segment of a bristle neuron; **b** axon of a bristle neuron; **c**, **d** photoreceptor cells; **e** 2° or 3° pigment cell; **f** cone cells. **a′**–**f′** Schemes highlight decorated MTs, with counterclockwise decorated MTs (CCW; minus end facing the observer) presented in grey, and clock-wise decorated MTs (CW; plus end facing the observer) shown in blue. Arrowheads, adherens junction; N, nucleus. Bar, 0.2 µm. **g**–**g″″″** Analysis of MT polarity in the various cell types of the eye. *n* indicates the number of decorated MTs scored. Schemes on the right or above the panels present the preferential MT orientation in the various cells, according to the results of analysis. The arrow indicates the centriole position in bristle neurons

The results of the hook assay are presented in Fig. 6. In bristle neurons, all decorated MTs curved in clockwise direction in the outer dendritic segment above the basal body and in counterclockwise direction in the cell portion below the basal body, indicative of MT minus ends being oriented towards the basal body in both cell regions. In all other cell types analyzed, MTs had preferentially (95–99%) counterclockwise hooks. Notably, this orientation was observed both distally and proximally of the nuclei in PRCs and 2°/3° PCs. The low percentage of clockwise MTs within PRCs (~ 5%; Fig. 6c, c′, g′, g″″) resided close to adherens junctions. In conclusion, the majority of MTs in PRCs, PCs, and cone cells had their minus end oriented towards the distal end of the cell throughout the entire cell length.

### Posttranslational modifications of microtubules

Tubulin is subjected to diverse posttranslational modifications (PTMs) that affect interactions with microtubule-binding proteins (MAPs) and that are indicative of differences in MT stability (Janke and Bulinski 2011; Janke 2014; Song and Brady 2015; Park and Roll-Mecak 2018). Prominent modes of PTMs on non-ciliary MTs are acetylation of α-tubulin at a conserved lysine (K40), detyrosination/tyrosination of the α-tubulin C-terminal end, and glutamylation on a glutamate γ-carboxyl group in the C-terminal region of α- and β-tubulin. The *Drosophila* genome contains 4 α-tubulin and 4 β-tubulin genes, and these tubulin isotypes exhibit differences in their PTM sites (Nielsen et al. 2010). FlyAtlas data (Robinson et al. 2013; https://motif.mvls.gla.ac.uk/flyatlas) indicate that *αTub84B* (α1 tubulin), *βTub56D* (β1 tubulin), and *βTub97EF* (β4 tubulin) are expressed at high level in the adult eye. Of these, α1 tubulin has acetylation and detyrosination sites, and α1 and β1 have glutamylation sites (Hoyle et al. 2008; Nielsen et al. 2010; Jenkins et al. 2017).

**Fig. 7:**
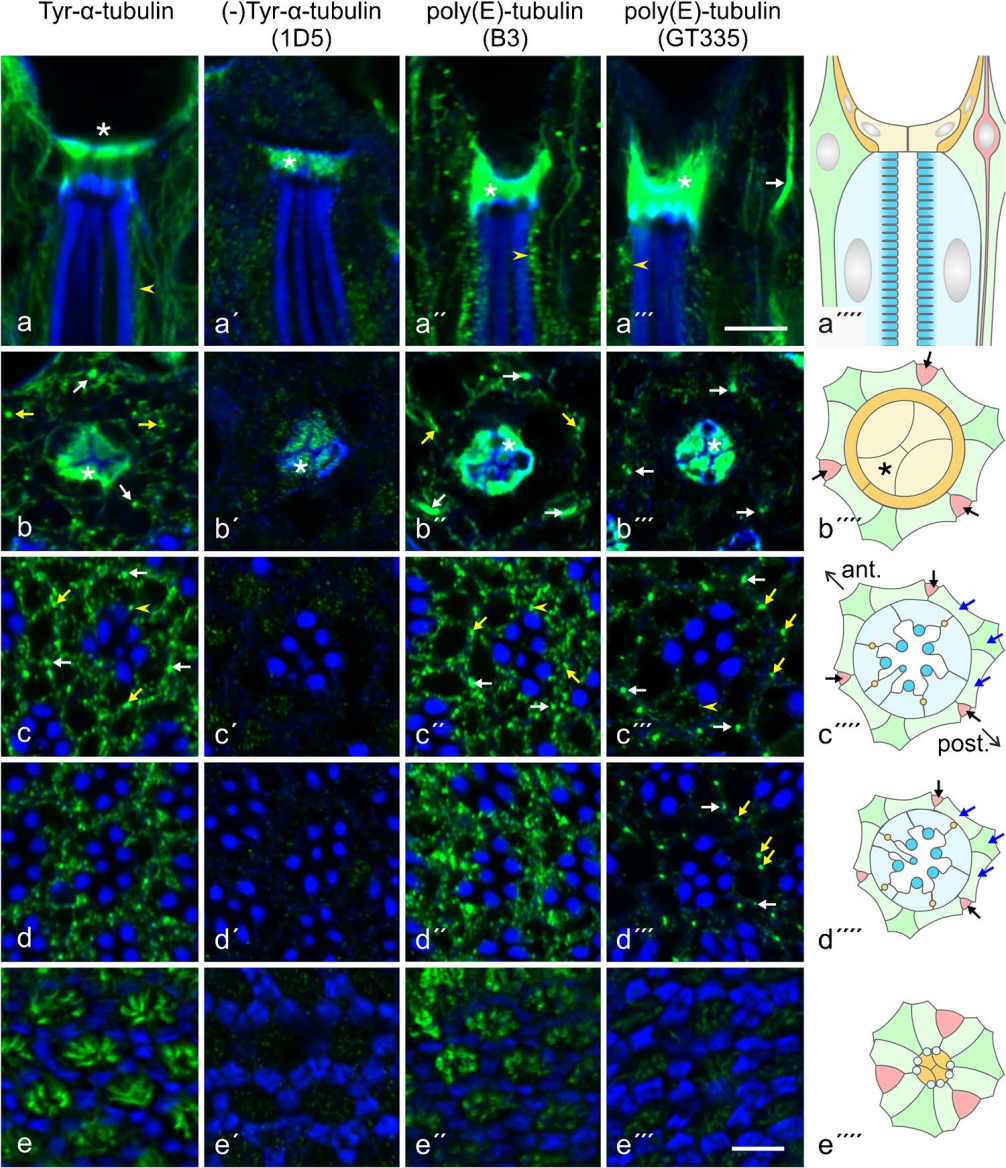
Distribution of posttranslational modifications (PTMs) of tubulin in the *Drosophila* eye. **a**–**a″″** The distal portion of ommatidia in longitudinal view. **b**–**b″″**, **c**–**c″″**, **d**–**d″″**, **e**–**e″″** Cross-sections at the level of cone cells (**b**–**b″″**), R7 cells (**c**–**c″″**), R8 cells (**d**–**d″″**) and of the retinal floor (**e**–**e″″**). Asterisks, cone cells; white arrows, bristle neurons; yellow arrows, MT bundles in pigment cells; arrowheads, MTs close to the rhabdomere base in photoreceptor cells. Note that Tyr-α-tubulin and B3 immunoreactivity have a distribution similar to β-tubulin. Immunoreactivity for (-)Tyr-α-tubulin is highly enriched in cone cells. GT335 immunoreactivity is enriched in bristle neurons (white and black arrows), in cone cells (asterisks), and in MT bundles within pigment cells (yellow and blue arrows). Color coding of schematic drawings (**a″″**–**e″″**) corresponds to schemes in Fig. 1. Bar, 5 µm

Using antibodies against posttranslationally modified tubulins, we have examined the presence and distribution of PTMs in the adult eye. By Western blot analysis, acetylated α-tubulin (Ac-α-tubulin), detyrosinated α-tubulin ( (-)Tyr-α-tubulin), and tyrosinated α-tubulin (Tyr-α-tubulin) were detected in the eye (Supplementary Material, Fig. S4a). Antibodies B3 and GT335, both probes for polyglutamylated tubulin (poly(E)-tubulin), provided different results on Western blots. Whereas B3 identified a band that co-migrated with α-tubulin, GT335 identified two bands, one of them co-migrating with α-tubulin and the other, more intense band with an electrophoretic mobility similar to β-tubulin (Supplementary Material, Fig. S4a, d). These results suggest that both α- and β-tubulin are polyglutamylated in the adult eye, unlike the brain which has this posttranslational modification exclusively on α-tubulin (Supplementary Material, Fig. S4c; Bobinnec et al. 1999; Devambez et al. 2017). To obtain information on the length of the glutamate chains, we used antibody 1D5 that was raised against (-)Tyr-α-tubulin but also reacts with side chains of at least 3 glutamyl units (Rüdiger et al. 1999). In order to distinguish between 1D5 immunoreactivity being due to detyrosination or polyglutamylation, homogenized retinae were incubated on ice to induce MT depolymerization and tubulin tyrosination, and the homogenate was then ultracentrifuged to pellet remaining MTs (Supplementary Material, Fig. S4b). The absence of tubulin in the pellet fraction demonstrates that MT depolymerization was complete. The presence of B3 immunoreactivity in the supernatant suggests that glutamylation was retained during cold treatment. The absence of 1D5 immunoreactivity in the supernatant suggests that (1) re-tyrosination of depolymerized α-tubulin was complete and (2) there were no poly-E side chains with at least 3 glutamyl units. Further support for the exclusive reactivity of 1D5 with (-)Tyr-α-tubulin was provided by analysis of developmental changes in PTM pattern (Supplementary Material, Fig. S5). Between late pupal development and early adult life, reactivity with anti-Tyr-α-tubulin and 1D5 changed in a reverse mode, with anti-Tyr-α-tubulin reactivity decreasing and 1D5 reactivity rising, indicative of detyrosination of some α-tubulin during this period. Reactivity with B3, however, remained constant demonstrating that the increase in 1D5 reactivity was not the result of polyglutamylation. Finally, the different immunofluorescence pattern obtained with 1D5 (Fig. 7a′–e′) vs. B3 and GT335 (Fig. 7a″–e″, a′″–e′″), respectively, corroborates the conclusion that poly(E)-tubulin is not identified by 1D5 in the adult retina. It may thus be deduced that antibody B3 identifies mono/bi-glutamylated α-tubulin, antibody GT335 seems to detect preferentially mono/bi-glutamylated β-tubulin, whereas antibody 1D5 detects only (-)Tyr-α-tubulin in the adult *Drosophila* eye.

By immunofluorescence microscopy, distribution of Ac-α-tubulin (Fig. S1), Tyr-α-tubulin (Fig. 7a–e), and B3 immunoreactivity (Fig. 7a″–e″) were indistinguishable from that of β-tubulin (Fig. 1), suggesting that all MT systems in the adult eye contain acetylated, tyrosinated, and mono/bi-glutamylated α-tubulin. (-)Tyr-tubulin was detected almost exclusively in cone cells (Fig. 7a′, b′). GT335 bound, in addition to cone cells, to bristle neurons and to, at the maximum, nine additional structures between neighboring retinulae, likely MT bundles in the 2°/3° PCs (Fig. 7a′″–e′″). Other MTs in PRCs and PCs displayed weak reactivity for GT335. This result indicates that mono/bi-glutamylated β-tubulin is enriched in cone cells, bristle neurons, and MT bundles of 2°/3° PCs.

## Discussion

### The MT system of photoreceptor cells

The present study has examined the arrangement of MTs in the various cell types that constitute the adult *Drosophila* compound eye. Particular focus was on the PRCs as these cells perform the primary function of the eye, to sense light and to translate the stimulus into an electrical signal. In addition, the PRCs are used as a model system for the analysis of protein targeting and establishment of cell polarity (Tong et al. 2011; Schopf and Huber 2017).

Our results demonstrate that photoreceptors have ncMTOCs associated with their plasma membrane at their very distal end, viz. at distinct sites of adhesion to cone cells. This conclusion is based on several complementary findings: (1) Confocal microscopy has visualized MT arrays that emanate from membrane-associated foci at the distal end of PRCs. (2) Electron microscopy has confirmed the presence of MTs that are inserted with one end in an electron matrix at adherens sites of PRCs to cone cells. (3) The spectraplakin Shot, a MT minus-end anchor on membranes (Nashchekin et al. 2016; Zheng et al. 2020), is localized to the plasma membrane in this cell region. (4) When the minus-end-transporting motor Nod::βgal was expressed in neuronal cells of the eye, it became enriched at the very distal end of the PRCs.

The finding that MTs originate at ncMTOCs is not unexpected since PRC precursors disassemble their centriole during the larval and early pupal stage (Riparbelli et al. 2018), and no centrosomal structures can be detected in adult PRCs (Mogensen et al. 1993; Riparbelli et al. 2018; present study). The organization of the MT system in adult PRCs is therefore similar to that in larval PRC precursors. Despite a centriole being still present at the larval stage, this structure does not serve as MTOC but MTs rather extend from an electron-dense plaque at the distal tip of the cell in proximal direction (Riparbelli et al. 2018). However, in larval PRC precursors but not in adult PRCs, the membrane area at the distal tip represents the apical, microvilli-bearing membrane. Since the apical microvilli-bearing surface is shifted by 90° onto the side of the PRCs at the beginning of pupal life (Longley and Ready 1995; Tepass and Harris 2007), the ncMTOC becomes spatially separated from this membrane domain during PRC morphogenesis.

MTs in the PRCs are aligned in distal–proximal direction and, with few exceptions, have their minus end oriented towards the distal cell end, irrespective of whether MTs reside below or above the nucleus. Such a unipolar MT organization supports anterograde transport of axonal and synaptic components via plus-end-directed MT motors. This is in line with the finding that functional defects in Milton, an adaptor protein for the plus-end-directed MT motor kinesin-I on mitochondria, cause a severe defect in the transport of mitochondria towards the synapse (Stowers et al. 2002; Górska-Andrzejak et al. 2003; Glater et al. 2006). Synapse structure and the accumulation of synaptic vesicles are only mildly effected by *milton* mutations, suggesting that MT-dependent anterograde transport of membranes other than mitochondria may either use different adaptor proteins for kinesin-I and/or other kinesins.

By both electron microscopy and immunofluorescence microscopy, we were unable to detect horizontally oriented MTs, extending from the cell body to the rhabdomere base. This cell area below the rhabdomere is dominated by the presence of actin filaments that project from the microvillar bases into the cytoplasm (Arikawa et al. 1990; Xia and Ready 2011). A similar organization of the cytoskeleton in the subrhabdomeric area, with an extensive system of actin filaments but no MTs extending from the rhabdomere base into the cytoplasm, has been described for other insect PRCs (Baumann 1992; Stürmer et al. 1995). It may thus be suspected that active material transport towards and away from the rhabdomere occurs exclusively in an actomyosin-dependent manner. Indeed, rhodopsin transport and pigment granule movement to the rhabdomere is impaired in *Drosophila* myosin-V mutants (Li et al. 2007; Satoh et al. 2008). The finding that mutations in *Glued* also produce defects in rhabdomere structure (Fan and Ready 1997), however, seems to contradict the above model. Glued is a component of the dynactin complex that links cytoplasmic dynein to cargos. On the same line, an RNAi screen demonstrated that cytoplasmic dynein and kinesin-I support apical transport of rhodopsin and of the proteoglycan Eyes shut, an extracellular matrix component secreted at the apical membrane (Laffafian and Tepass 2019). How can these observations be reconciled with the absence of MTs in an orientation required for MT-dependent transport from the cytoplasm towards the apical membrane? We suppose that newly synthesized proteins destined for the apical membrane are sorted at the TGN and then transported towards the target domain in a 2-step process with participation of the MT and the F-actin system. Carrier vesicles may be distributed along the cell body on longitudinally arranged MTs in a bidirectional mode until they hop on actin filaments for myosin-dependent transport to the rhabdomere. Alternatively, defects in the MT system may influence the organization of the F-actin system and thus harm actomyosin-dependent vesicle transport in an indirect mode. Notably, there is a multitude of interactions between MT and F-actin systems (Dogterom and Koenderink 2019), and mutations in MAPs, including dynein heavy chain, have been reported to disrupt the F-actin system in *Caenorhabditis elegans* (Gil-Krzewska et al. 2010).

### Organization of the MT system in pigment cells

Although the morphology of the adult *Drosophila* eye has been studied extensively, the MT system in the 2° and 3° PCs has not been described yet. Both immunofluorescence microscopy and electron-microscopic imaging have visualized two sets of MTs in each 2° and 3° PC, single MTs distributed over the entire cross-section of the cell, and a bundle of MTs, nearly hexagonally packed with a few interleaved actin filaments. In both sets, MTs have a unipolar orientation, with the minus end facing the distal cell end and the MT plus end the retinal floor. We suppose that these two MT sets serve different functions. This hypothesis is corroborated by the finding that single MTs and bundled MTs differ in PTMs, suggesting an association with different MAPs (see below).

2° and 3° PCs extend over the entire thickness of the retina, from the cornea down to the basal lamina, with the nucleus positioned at the level of the cone cells. The set of individual MTs may provide tracks for the transport of membranous organelles and macromolecular components from the perinuclear area towards the proximal and distal cell area or vice versa. Unfortunately, studies on the effects of mutations in genes encoding for MT motors or adaptor proteins were focused on PRCs and did not analyze whether PC morphology and/or functions are also impaired.

MT bundles may have a primarily mechanical supportive role, as do similar MT systems in other cell types. In *Drosophila* wing epidermal cells and in inner ear supporting cells, closely packed MTs in unipolar orientation and with actin filaments in parallel extend along the cell’s long axis and are supposed to have a stiffening effect (Slepecky and Chamberlain 1983; Mogensen and Tucker 1988; Mogensen et al. 1989, 2002). In the *Drosophila* eye, PCs may assume a mechanically supportive role not only to maintain retinal architecture but also as an abutment for the contractile force that PRCs exert during the phototransduction process (Hardie and Franze 2012).

### Posttranslational modifications of tubulin in the *Drosophila* eye

**Fig. 8:**
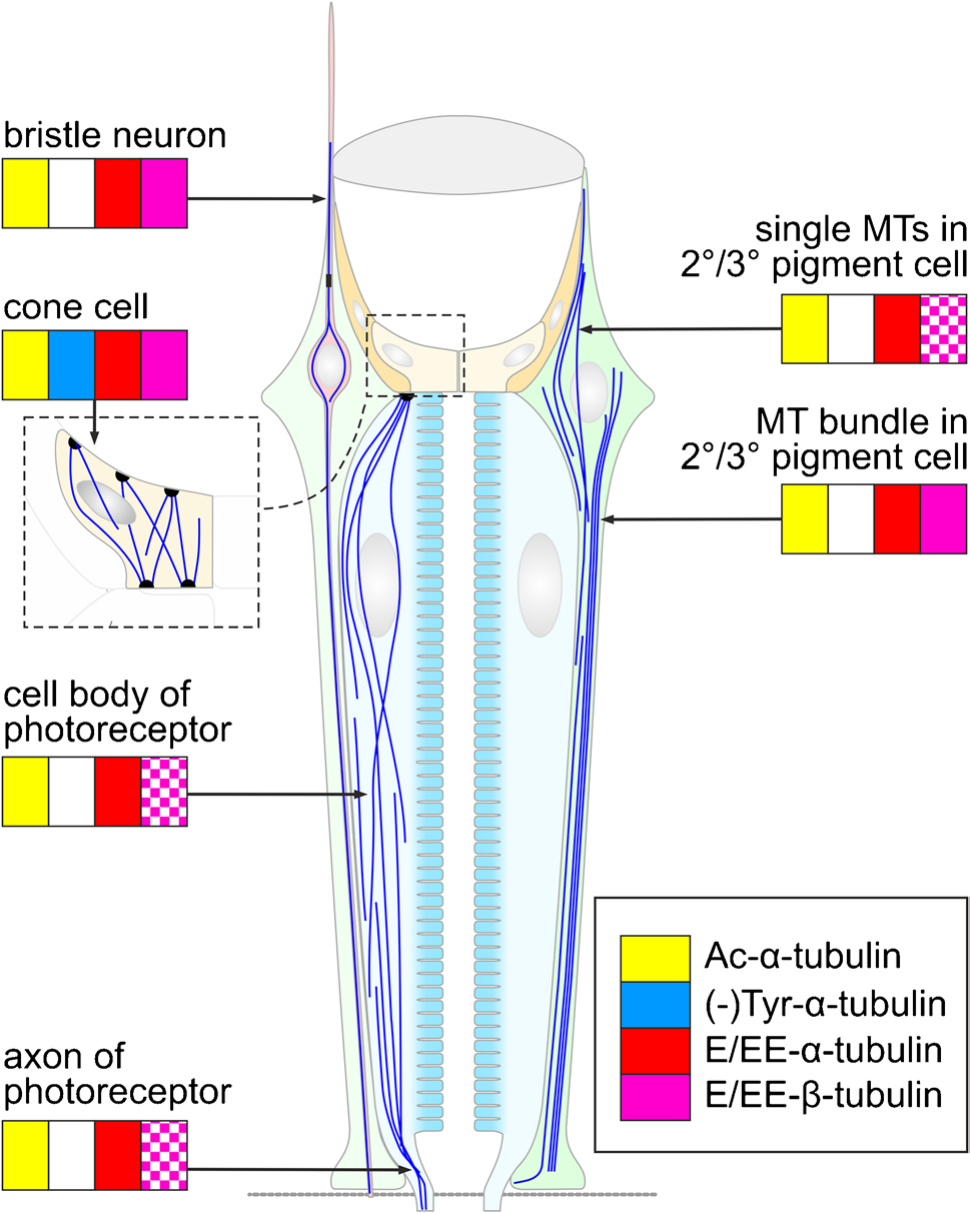
Schematic presentation of the distribution of posttranslational modifications on tubulin in the *Drosophila* eye. Tiled fillings indicate relatively weak labeling. Ac-α-tubulin, acetylated α-tubulin; (-)Tyr-α-tubulin, detyrosinated α-tubulin; E/EE-α-tubulin, mono/bi-glutamylated α-tubulin; E/EE-β-tubulin, mono/bi-glutamylated β-tubulin

Differences in PTMs between the various MT populations in the eye (Fig. 8) are an indication of distinctive physical properties and/or functions. The presence of Ac-α-tubulin in the adult eye was demonstrated by Western blots. This is consistent with the finding that both tubulin acetyltransferase Atat (CG3967), the enzyme that transfers an acetyl group to a conserved K40 on α-tubulin (Niu et al. 2023), and histone deacetylase 6 (HDAC6), the enzyme that removes this PTM (Yan et al. 2018), are expressed in this tissue (https://motif.mvls.gla.ac.uk/flyatlas). Immunofluorescence microscopy has demonstrated further that acetylation is present at all MT populations in the eye. Actually, we did not observe any differences in labeling pattern obtained with anti-β-tubulin and anti-Ac-α-tubulin, except for a lower background staining with the latter antibody. This is not surprising since acetylation occurs exclusively on α-tubulin incorporated in MTs (Janke and Montagnac 2017) whereas anti-α-tubulin may also detect non-polymerized α-tubulin in the cytosol. Anti-Ac-α-tubulin thus serves as an excellent marker for the entire MT system in the adult eye. Acetylation of α-tubulin has been shown to protect MTs against breakage during mechanical stress and is hence indicative of long-lived MTs (Janke and Montagnac 2017; Xu et al. 2017). It may be concluded that the various MT systems in the adult eye, all of them extending in distal–proximal direction, are mechanically stabilized to sustain shearing forces.

Since α1 tubulin with a C-terminal tyrosine is the only α-tubulin isotype expressed in the eye, theoretically all MT systems in the eye could be substrates for detyrosination. Antibody 1D5, a probe for (-)Tyr-α-tubulin, however, bound only to cone cells. Conversely, antibody TUB-1A2 against Tyr-α-tubulin labeled all MT populations in the eye. Detyrosination of α-tubulin thus seems to occur exclusively on cone cell MTs, and this reaction happens late in pupal development (Fig. S5). Vasohibins and tubulin metallocarboxypeptidase TMCP1 were identified as the enzymes that perform tubulin detyrosination in vertebrates (Nieuwenhuis and Brummelkamp 2019; Nicot et al. 2023). Since no vasohibin and TMCP homologs were found in hexapods (Sanchez-Pulido and Ponting 2016; Nicot et al. 2023), the enzyme executing this reaction in *Drosophila* is still unidentified, and it must remain open whether selective expression of the tubulin-detyrosinating enzyme accounts for the restriction of this PTM to cone cells.

Glutamylation can occur on the C-terminal domain of α- and β-tubulin, with the length of the side chain varying between one to 20 glutamyl units (Janke et al. 2008). In the adult *Drosophila* eye, both α- and β-tubulin seem to be glutamylated, and glutamyl side chains seem to be confined to a length of 1 and/or 2 units. Whereas α-tubulin is mono/bi-glutamylated in all MT systems in the eye, mono/bi-glutamylated β-tubulin has a differential distribution, being enriched in bristle neurons, cone cells, and in bundled MTs in PCs (Fig. 8). In the adult *Drosophila* nervous system, in contrast, glutamylation occurs predominately on α-tubulin, and glutamyl chains may become longer than 2 residues (Bobinnec et al. 1999; Devambez et al. 2017). DmTTLL5 has been identified as a tubulin glutamylase in *Drosophila* tissues (Devambez et al. 2017; Bao et al. 2023), and this enzyme is also expressed in the eye, although at a lower level than in the brain (https://motif.mvls.gla.ac.uk/flyatlas). Since TTLL5 initiates glutamate chains specifically on α-tubulin (Devambez et al. 2017), it may be expected that additional, yet unidentified tubulin glutamylases are present in the eye and act on β-tubulin.

The PTM pattern of α- and β-tubulin, along with the expression of different tubulin isotypes, was proposed to represent a code that is translated by differential binding of effector proteins into specific functions (Verhey and Gaertig 2007; Yu et al. 2015). Both detyrosination/tyrosination and glutamylation occur on the C-terminal domain of tubulin that is localized on the outer MT surface and involved with the binding of motor proteins and immobile MAPs (Janke et al. 2008; Yu et al. 2015; Janke and Montagnac 2017; Nieuwenhuis and Brummelkamp 2019). The above differences in PTMs between the various MT systems in the adult eye may thus influence interactions with MAPs and lend different properties and functions to different MT systems. MT detyrosination, for instance, has been demonstrated to decrease dynein-mediated motility (McKenney et al. 2016). Similarly, the length of poly-glutamyl side chains on tubulin influences the binding and activity of MT motors and the interaction with structural MAPs (Boucher et al. 1994; Larcher et al. 1996; Sirajuddin et al. 2014). It is to be assumed that glutamylation of β-tubulin on MTs in pigment cells creates the prerequisite for binding a yet unidentified MAP, which leads to MT bundling and/or association with actin filaments. Unfortunately, the cellular and subcellular expression pattern of MAPs in the adult *Drosophila* eye has not yet been analyzed, and it thus remains unknown whether the distribution of any MAP correlates with the PTM pattern.

## Supplementary Information

Below is the link to the electronic supplementary material.

Supplementary file1 (PDF 1.08 MB)

## Data Availability

All relevant data are included in the manuscript. Raw data are available from the corresponding author upon reasonable request.
