# Supplementary material for: Spatial arrangement, polarity, and posttranslational modifications of the microtubule system in the Drosophila eye
Source: Cell Tissue Res. 2024 Aug 17;398(2):123–37. doi: 10.1007/s00441-024-03914-6 (PMC11525301; doi:10.1007/s00441-024-03914-6)
Supplement: Supplementary file 1 — Supplementary file1 (PDF 1.08 MB) [file 441_2024_3914_MOESM1_ESM.pdf]

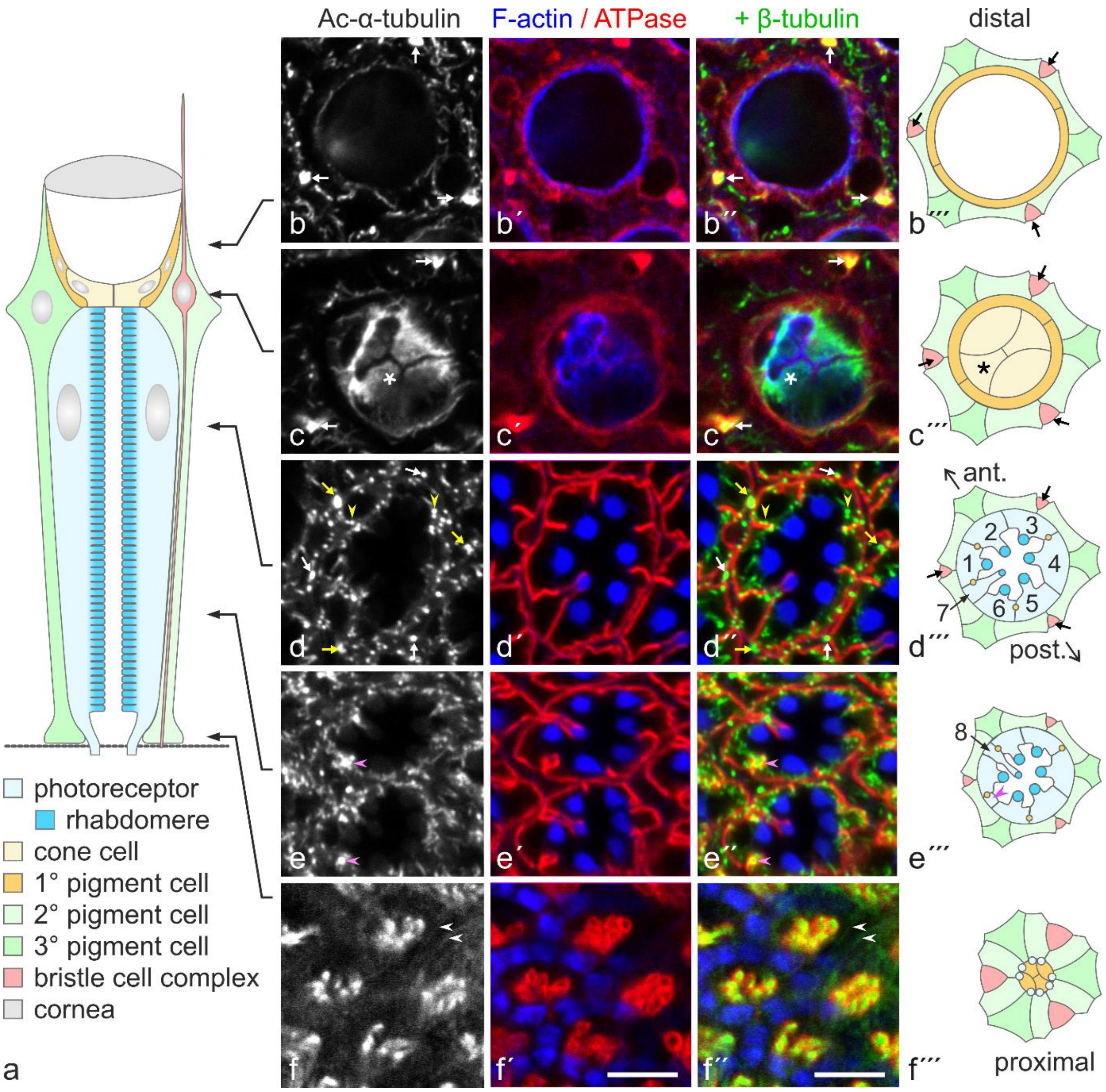

Supplementary Fig. S1 Immunofluorescence localization of microtubules (MTs) in the adult *Drosophila* eye. Cryo-sections at various levels (as indicated in the schematic drawing in **a**) were labeled with anti-acetylated-α-tubulin (Ac-α-tubulin; green in **b´´-f´´**), AlexaFluor 488 phalloidin (F-actin; blue in **b´-f´**, **b´´-f´´**) and anti-Na,K-ATPase (ATPase; red in **b´-f´**, **b´´-f´´**). **b´´´-f´´´** Schematic drawings indicate the cell pattern according to the level of the individual cryo-section; anterior-posterior orientation is indicated (ant. / post.) and corresponds to the orientation of the sections. For simplicity, the bristle cell complex is not subdivided into its individual cells (bristle neuron, supporting cells) in the schematic drawings. Na,K-ATPase labeling outlines bristle neurons (**b´´**, **c´´**, white arrows), cone cells (**c´´**) and photoreceptor cells (**d´´**, **e´´**, **f´´**). Ac-α-tubulin in cone cells, (asterisks in **c**, **c´´**) colocalizes with F-actin. Bristle neurons (white arrows in **b´´-d´´**; black arrows in **b´´´-d´´´**) are intensely labeled for Ac-α-tubulin. Several tubulin-positive structures between the photoreceptors (yellow arrows in **d**, **d´´**) and of a similar intensity to bristle neurons indicate MT bundles in 2°/3° pigment cells. Dot-like Ac-α-tubulin-positive structures (yellow arrowheads) indicate MTs in longitudinal orientation in cross-sectioned photoreceptors. Pedicels of 2°/3° pigment cells at the retinal floor have MTs en-face-view (white arrowheads in **f**, **f´´**). Magenta arrowheads in **e**, **e´´**, **e´´´**, R7 axons. Bar, 5 µm

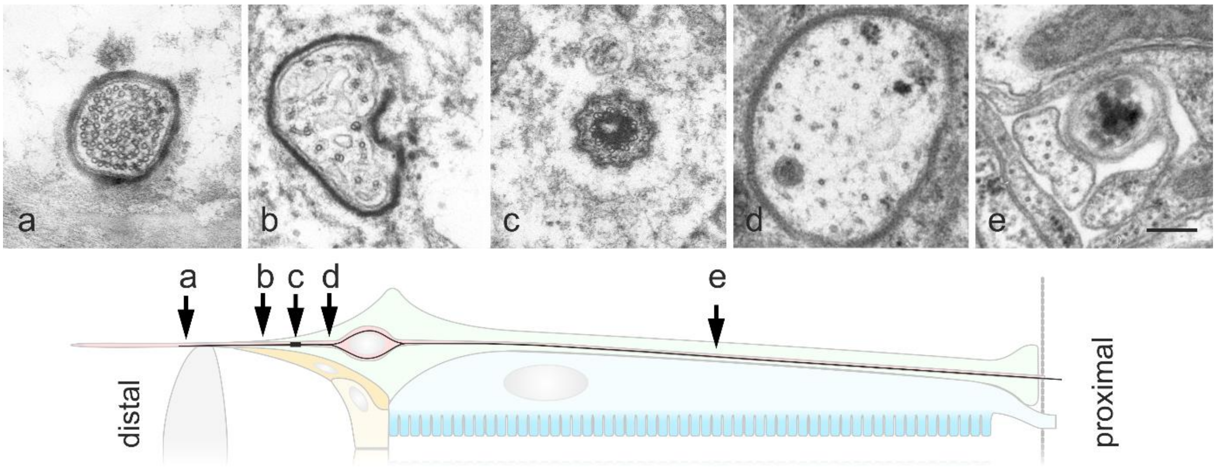

Supplementary Fig. S2 The microtubule system in bristle neurons. **a-e** Electron micrographs of cross-sections at various levels of the sensory bristle neuron. Sectioning planes are indicated in the scheme below. **a** Tubular body in the distal portion of the dendritic outer segment; **b** proximal portion of dendritic outer segment; **c** basal body; **d** dendritic inner segment; **e** axon. Bar, 0.2 µm

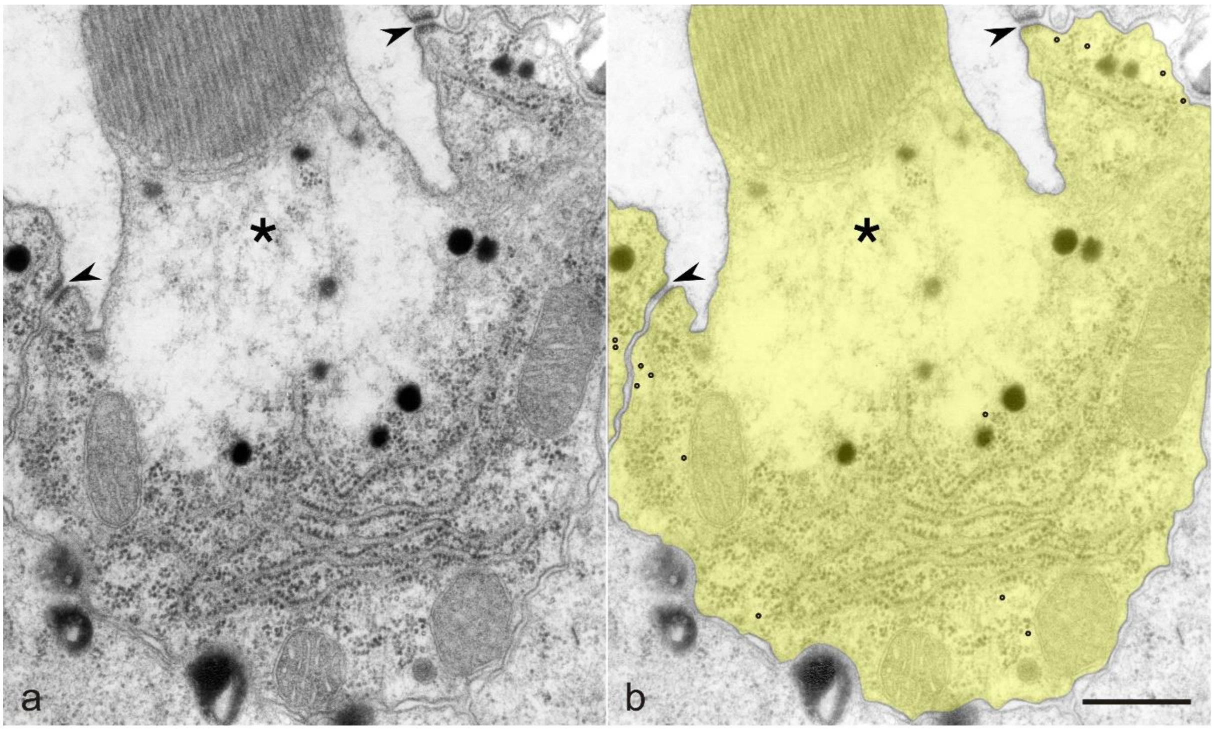

Supplementary Fig. S3 Microtubules in photoreceptor cells. **a** Electron micrograph of a cross-section through photoreceptor cells. **b** Position of MTs highlighted in photoreceptor cells (yellow). Note the absence of MTs in the subrhabdomeric cytoplasm (asterisk). Arrowheads, adherens junction. Bar, 0.5 µm

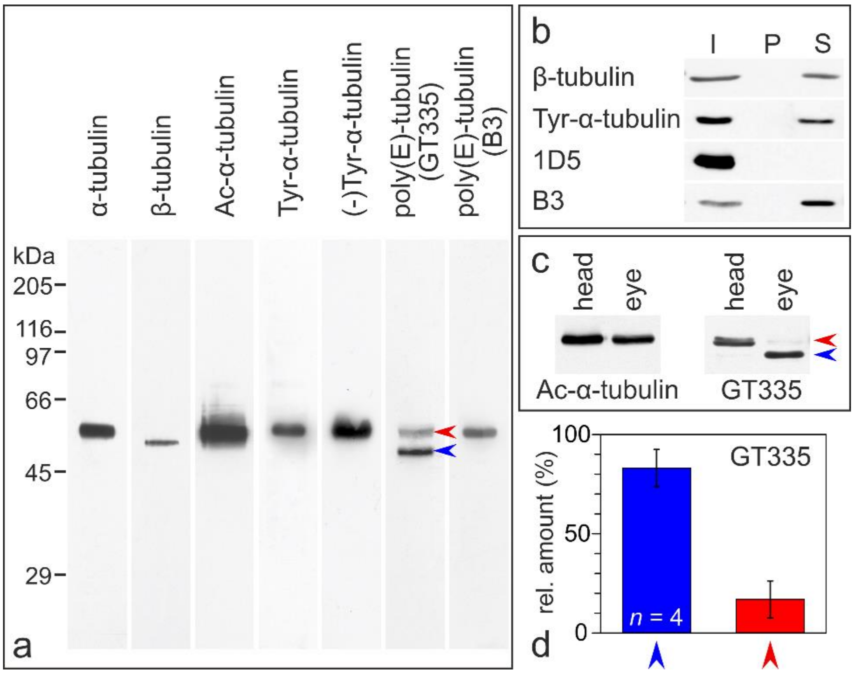

Supplementary Fig. S4

a Western blot analysis of *Drosophila* eye with antibodies against posttranslationally modified tubulin. Anti-α-tubulin and anti-β-tubulin each label a single band in Western blots, with the band identified by anti-β-tubulin migrating slightly faster. Antibodies against acetylated α-tubulin (Ac-α-tubulin), tyrosinated α-tubulin (Tyr-α-tubulin), detyrosinated α-tubulin (1D5, (-)Tyr-α-tubulin) and antibody B3 against polyglutamylated tubulin (poly(E)-tubulin) each identify a single band that comigrates with α-tubulin. Antibody GT335 against poly(E)-tubulin labels two bands that may represent α-tubulin and β-tubulin (red and blue arrowheads).

b Immunoreactivity with anti-(-)Tyr-α-tubulin (1D5) and anti-poly(E)-tubulin B3 after depolymerization of microtubules (Methods, see below). 1D5 does not identify depolymerized tubulin, but anti-poly(E)-tubulin B3 does, supporting the notion that antibody 1D5 identifies (-)Tyr-α-tubulin but not poly(E)-tubulin in *Drosophila* eyes. I, Input (before cold treatment); P, pellet after cold treatment to depolymerize microtubules; S, supernatant after cold treatment.

c Polyglutamylation pattern of tubulin differs between eye and brain. Note that anti-poly(E)-tubulin GT335 labels (at least) two bands in both head and eye, but the bands differ by their relative intensities (red and blue arrowheads).

d Quantitative analysis of the intensity of the two bands labeled by anti-poly(E)-tubulin GT335. Data are presented as mean ± SEM, $n = 4$. The lower band (blue arrowhead) contains 83.1% of the staining intensity, the upper band (red arrowhead) 16.9%. We note, however, that the relative distribution was quite variable between preparations (**a, c**), providing the possibility that the upper band is at least partially due to contamination of the preparation by brain tissue.

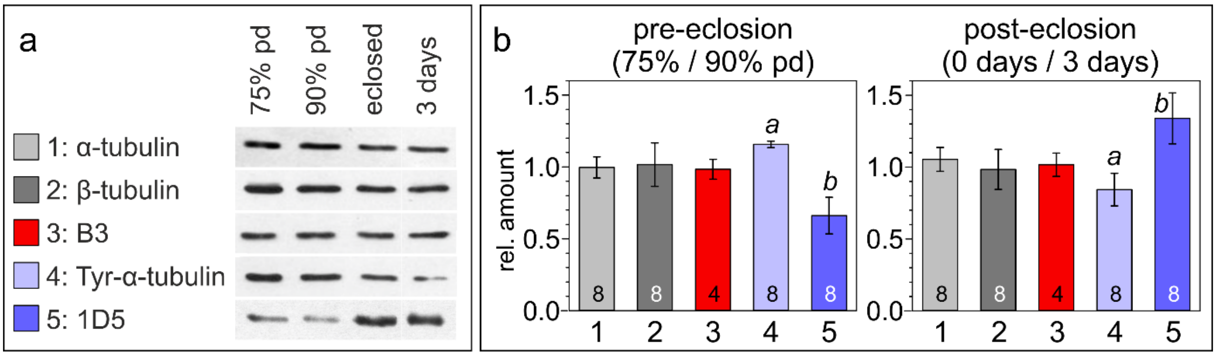

Supplementary Fig. S5 Posttranslational tubulin modifications during eye development. Labelings for (-)Tyr-α-tubulin (1D5; #5) and Tyr-α-tubulin (#4) change in a reverse mode during eye development, whereas reactivities with anti-α-tubulin (#1), anti-β-tubulin (#2) and anti-poly(E)-tubulin B3 (#3) remain constant, supporting the notion that 1D5 identifies specifically (-)Tyr-α-tubulin. %pd, stage of pupal development; eclosed, 0 day post-eclosion; 3 days, 3 days post-eclosion. **a** Representative Western blots. **b** Quantitative analysis. Data are presented as mean ± SEM. The number in each bar represents the number *n* of experiments (e.g. 8 = 4 x 75% pd + 4 x 90% pd). Labeling for Tyr-α-tubulin decreases significantly (*a*, P = 0.028; Mann-Whitney Rank Sum test), labeling with antibody 1D5 increases significantly (*b*, P = 0.010) between late pupal development (75% pd and 90% pd) and early adulthood (0 day and 3 days post-eclosion).

Methods: S4a, c *Drosophila* eyes (**a, c**) and heads (**c**) were dissected and homogenized in reducing sample buffer (Carl Roth, Karlsruhe, Germany). The preparations were heated to 95°C for 5 min and centrifuged for 10 min at 16,000xg to remove non-solubilized material. Equal amounts of protein were loaded per lane on 10% SDS polyacrylamide gels and immunoblotted. **S4b** To induce MT depolymerization by cold treatment, *Drosophila* heads were homogenized in 50 mM 2-(N-morpholino)ethanesulfonic acid, 1 mM $MgCl_2$, 2 mM EGTA, 2% protease inhibitor cocktail, pH 6.8 at 4°C, incubated for 30 min at 0°C, and centrifuged for 10 min at 1,800xg at 4°C to pellet cell fragments and nuclei. The supernatant was centrifuged for 30 min at 126,000xg at 4°C to pellet remaining microtubules. Proteins of the resulting pellet and supernatant were then separated on SDS gels and immunoblotted. **S5** *Drosophila* eyes were isolated from animals of different developmental stages and homogenized in reducing sample buffer. Equal amounts of protein were loaded per lane, with one sample of each 75% pd, 90% pd, eclosed imagines and 3-days-old imagines loaded on the same gel and treated in parallel during immunolabeling and imaging. Staining intensities were quantified and normalized to the mean of the four developmental stages processed in parallel.
